# 3D Printing in the Design of Potentiometric Sensors: A Review of Techniques, Materials, and Applications

**DOI:** 10.3390/s25164986

**Published:** 2025-08-12

**Authors:** Aleksandra Zalewska, Nikola Lenar, Beata Paczosa-Bator

**Affiliations:** Faculty of Materials Science and Ceramics, AGH University of Krakow, Mickiewicza 30, PL-30059 Krakow, Poland; azalewska@agh.edu.pl

**Keywords:** 3D printing, ion-selective electrodes, electrochemical sensors, rapid prototyping, sensor miniaturization

## Abstract

The integration of 3D printing into the development of potentiometric sensors has revolutionized sensor fabrication by enabling customizable, low-cost, and rapid prototyping of analytical devices. Techniques like fused deposition modeling (FDM) and stereolithography (SLA) allow researchers to produce different sensor parts, such as electrode housings, solid contacts, reference electrodes, and even microfluidic systems. This review explains the basic principles of potentiometric sensors and shows how 3D printing helps solve problems faced in traditional sensor manufacturing. Benefits include smaller size, flexible shapes, the use of different materials in one print, and quick production of working prototypes. However, some challenges still exist—like differences between prints, limited chemical resistance of some materials, and the long-term stability of sensors in real-world conditions. This paper overviews recent examples of 3D-printed ion-selective electrodes and related components and discusses new ideas to improve their performance. It also points to future directions, such as better materials and combining different manufacturing methods. Overall, 3D printing is a powerful and growing tool for developing the next generation of potentiometric sensors for use in healthcare, environmental monitoring, and industry.

## 1. Introduction

In the modern world, chemical analysis is present in numerous fields, including industry, medicine, and environmental studies. Rapid development demands that scientists continue to develop new quantitative and qualitative analysis methods and improve parameters such as sensitivity and accuracy in existing methods [1]. Sciences focused on chemical sensors are developing intensively. Chemical sensors have gained popularity due to several features, including the capability for point-of-use (POU) measurements [2]. Modern designs enable the in situ measurement of analyte concentrations, eliminating the need for sampling. This is especially important for environmental monitoring, including water and soil pollution control. The quick availability of measurement results is crucial for analyses in diagnostics and patient health monitoring, facilitated by point-of-care (POC) devices [3].

In chemical sensors, electrochemical methods are most commonly used due to the relatively easy miniaturization of the measurement system [4]. This article focuses on sensor solutions based on potentiometry, which is used to determine the quantitative amount of an ionic analytes in test samples. Its advantages include high selectivity, high sensitivity, and a low detection limit. Such sensors are based on ion-selective electrodes, where the membrane is responsible for the sensor’s selectivity [5].

The primary goals in sensor development are to reduce production costs, accelerate manufacturing processes, and enable operation by individuals without specialized qualifications. One idea that has emerged over the years is using paper as a substrate for functional elements, a concept proposed in the 2000s by the Whitesides group [6]. Another emerging topic in the analysis of biological components is wearable sensors [7]. These devices incorporate miniaturized sensors that allow continuous measurement. They can take the form of watches, bands, or patches, making them ideal for wearable applications [8].

The advancement of sensors also involves attempts at miniaturization, which for potentiometric sensors required the elimination of the internal solution. This necessitated replacing it with a material capable of converting ionic to electronic conductivity [4].

The first solid contact electrodes known as CWE (Coated Wire Electrode) were constructed by R. W. Cattrall and H. Freiser in 1971. Their design consisted of metal wires coated with an ion-selective polymer membrane. Initially, these electrodes were capable of detecting calcium ions (Ca^2+^), and further improvements enabled the detection of other ions, which significantly contributed to the development of ion-selective electrodes [7].

A significant driving force for development was the discovery of the conductive properties of polymers by Hideki Shirakawa [9]. Polymers were first used as solid contacts in ion-selective electrodes by Lewenstam’s team in the early 1990s. They employed polypyrrole (PPy) as an intermediate layer between the ion-selective membrane and the electrical output, resulting in significantly improved stability compared to conventional wire electrodes (CWEs) [10,11,12].

This article focuses on the use of a Rapid Manufacture Method—3D printing for producing potentiometric sensors. Also known as additive prototyping, 3D printing enables rapid production and, consequently, near-instant testing and verification of the properties of the created object [13]. Three-dimensional printing is a technique that allows for creating physical objects layer by layer. This method is characterized by the quick production of components without the need to modify or build complex production lines, allowing for an almost immediate assessment of usability of the printed objects [14,15,16].

The article describes the most popular 3D printing methods and compiles information on producing individual elements of the sensor system. Potentiometric sensors could either be fully printed as ready-to-use devices or incorporate printed components into their design. The continuous emergence of new materials suitable for 3D printing consistently expands the range of additive methods, enabling the printing of not only sensor housing but also ion-selective electrodes or membranes. The primary goal of this article is to discuss the advancements and applications of 3D printing in the development of potentiometric sensors [2].

## 2. Fundamentals of Potentiometric Sensors

Potentiometric sensors are electrochemical devices that respond to the activity of specific ions in a solution by generating a measurable potential, which—under zero current conditions—is recorded between a reference electrode and a working electrode using a high-impedance voltmeter; the term ‘sensor’ may refer either to the working electrode alone or, more broadly, to the entire electrochemical cell. The potential developed at the working electrode arises from the selective interaction between the ion-selective membrane and the target ions in solution. This potential is fundamentally described by the Nernst equation, which mathematically relates the electrode potential *E* to the logarithm of the ion activity *a*:

(1) $$E=E^{0}+\frac{RT}{nF}\ln a$$

where *E*^0^ is the standard electrode potential, *R* is the gas constant, *T* is the temperature in kelvin, *z* is the charge of the ion, and *F* is the Faraday constant. The fundamental setup includes an ion-selective electrode (ISE) as the sensing element and a stable reference electrode, typically Ag/AgCl or calomel, immersed in the same electrolyte [17,18].

ISEs operate through a membrane that selectively interacts with a specific ion, generating a potential that varies according to the ion’s activity in the test solution. Depending on the application and target ion, the membrane can be glass (commonly used in pH sensors), crystalline (e.g., LaF_3_ for fluoride detection), or polymeric liquid membranes based on polyvinyl chloride (PVC) doped with ionophores (such as valinomycin for potassium). These membranes are designed to achieve a Nernstian response, which for monovalent ions at 25 °C equals approximately 59.16 mV per tenfold change in concentration To obtain a high-quality analytical signal, the working electrode must satisfy key requirements: it should exhibit high selectivity toward the target ion over interfering species, rapid and reproducible response time, minimal signal drift (stability), and an appropriate detection limit to quantify ions at relevant concentrations. These factors depend critically on the membrane composition, ionophore properties, and the physical and chemical stability of the sensor assembly [17,18,19].

Beyond classical ion-selective electrodes, potentiometric biosensors integrate biological recognition elements—such as enzymes, antibodies, or aptamers—into the sensor design. These biorecognition layers convert biochemical interactions into measurable electrochemical signals. A typical example involves enzyme-based sensors, such as glucose biosensors, where the enzymatic reaction generates or consumes protons or ions, thereby modifying the local potential sensed by a pH- or ion-selective membrane [20]. Immunosensors and aptamer-based sensors extend the concept further by enabling selective detection of proteins, small molecules, or even whole cells using specific binding interactions that alter the local ion environment near the transducer [21].

Classical materials used in potentiometric sensors include PVC for flexible membranes, plasticizers to control membrane viscosity, ionophores for selective ion binding, and lipophilic additives to improve ion-exchange kinetics. Traditional fabrication techniques involve solvent casting, drop-casting, or dip-coating membranes onto solid supports such as glassy carbon, platinum, or screen-printed electrodes. Internal filling solutions or solid-contact layers—such as conductive polymers (e.g., polypyrrole or poly(3-octylthiophene))—enable stable transduction of ionic signals into electrical output. Solid-contact ISEs are especially favored for miniaturized, wearable, or portable sensors due to their stability and lack of internal liquid junctions [22].

The evaluation of potentiometric sensors relies on several critical analytical parameters, each reflecting a different aspect of sensor performance. Sensitivity—often expressed as the slope of the calibration curve in mV per decade of ion activity—is a measure of how effectively the sensor converts changes in ion concentration into measurable potential changes. The detection limit defines the lowest ion activity detectable above the background noise. The linear range refers to the concentration interval over which the sensor response remains directly proportional to ion activity, while response time measures how quickly the sensor reaches a stable potential after exposure to the analyte. Selectivity, quantified by selectivity coefficients, assesses the sensor’s ability to discriminate the target ion from interfering species, and is especially critical in complex sample matrices; it is commonly evaluated following IUPAC guidelines using methods like the separate solution or fixed interference approaches. Stability of potentiometric response is described by a signal drift over time. Accuracy refers to how close a sensor’s measured value is to the true or accepted reference value. In potentiometric sensing, high accuracy means that the potential measured by the electrode reliably corresponds to the actual ion activity or concentration in the sample. It reflects the correctness of the measurement, rather than its consistency. On the other hand, reproducibility and repeatability reflect the sensor’s consistency and reliability in repeated measurements. These parameters are influenced by the sensor’s material properties, such as membrane composition (PVC, plasticizers, ionophores, lipophilic additives) and the quality of fabrication processes, which researchers optimize to enhance overall sensor performance [19,23].

Overall, potentiometric sensors offer significant advantages: they are inexpensive, simple to construct, provide rapid responses, and require minimal power. These characteristics make them suitable for a wide range of applications, including environmental monitoring, clinical diagnostics, industrial process control, and more recently, integration into wearable and point-of-care devices [20,21].

## 3. Rapid Prototyping—3D Printing

Starting in the 1980s, the rapid development of additive printing methods contributed to innovative solutions and advancements in different types of chemical sensors including potentiometric. Additive manufacturing uses a layer-by-layer technique to build objects, allowing for much faster prototype creation, which facilitates design evaluation and testing before introducing a product into mass production. In additive methods, 3D models are first created using computer-aided design (CAD) software, and 3D printers then construct 3D objects based on these models. CAD or CAM (computer-aided manufacturing) are advanced computer tools that supports both the design and production stages, commonly used to support processes from initial phases such as designing and simulating properties to actual printing and documentation. A CAD model contains information on the object’s geometry as well as parameters like topological constraints and mathematical relationships between dimensions [14,15,16].

**Figure 1:**
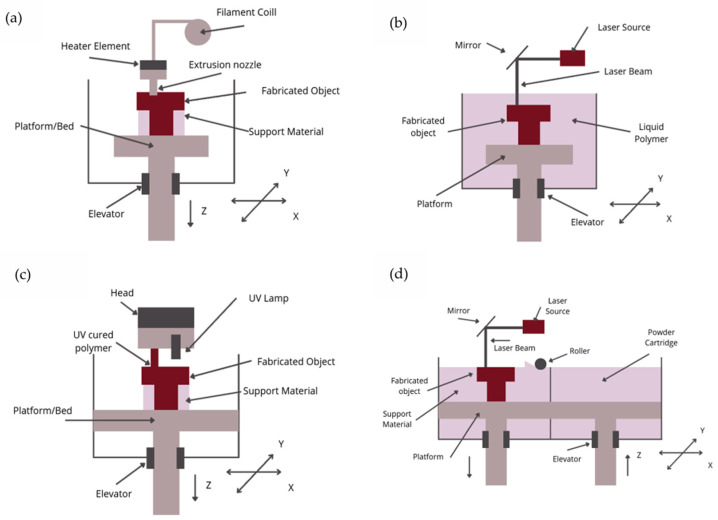
Schematic diagrams of most popular 3D printing techniques based on [26]: (**a**) Fused Deposition modeling–FDM; (**b**) Stereolithography -SLA; (**c**) Polyjet Process; (**d**) Selective laser sintering–SLS.

3D printing is a general term that encompasses various techniques used for the additive manufacturing of these designs. Different printing techniques, including inkjet printing, screen printing, stereolithography and fused deposition modeling (FDM), have been employed to simplify the production of potentiometric sensors, allowing for the elimination of several production stages, which were particularly time- and labor-intensive, especially in the production of ion-selective electrodes [2,24]. Some of the most popular types of 3D printing techniques are presented in Figure 1.

There are various types of 3D technology, which can be divided into 7 categories:

- Binder Jetting (BJ): A method in which a liquid binder is selectively applied to bond powdered materials;
- Directed Energy Deposition (DED): Techniques where thermal energy is used to melt materials layer by layer;
- Material Extrusion (ME): A method where material is extruded through a nozzle (also known as FDM—Fused Deposition Modeling);
- Material Jetting (MJ): Involves the selective deposition of material droplets, including photopolymers (Polyjet Process) and wax;
- Powder Bed Fusion (PBF): A 3D printing method where regions of a powder bed are fused using thermal energy; includes techniques like Selective Laser Sintering (SLS).
- Sheet Lamination (SL): An additive manufacturing process in which sheets of material are bonded to create the final product;
- Vat Photopolymerization (VP): A process in which an object is created from a liquid photopolymer in a vat and cured through light-activated polymerization; includes techniques like SLA–stereolitography [25].

Among these, the most commonly used 3D printing techniques in the construction of potentiometric sensors are FDM, SLA, PolyJet, and SLS, all of which are described in detail later in the article.

### 3.1. FDM—Fused Deposition Modeling

The most common and cost-effective 3D printing method is Fused Deposition Modeling (FDM). This method has been developed by S. Scott and L. Crump, which in 1980 started a company called Stratasys (Minnetonka, MN, USA) and in 1989, they filled in a patent application for a rapid prototyping method known as FDM, in which plastic filament or metal wire is heated in a nozzle and extruded. The application of the material is computer-controlled based on a previously prepared digital model. Stratasys developed whole systems for 3D printing using thermoplastics and printers [27]. The basic concept of the FDM manufacturing process involves melting the raw material and shaping it into new forms and structures [28]. FDM printers can be equipped with one or multiple extruders, allowing the use of several materials in a single print [14,15,16].

The material used in FDM is typically a thermoplastic polymer, selected based on the desired properties of the final product. It comes in the form of a filament, which is a thin thread of plastic on a spool with a diameter ranging from 1.75 to 2.85 mm. This filament is heated in the extruder’s hot end until it melts, then pushed through a nozzle and deposited onto the build platform. The nozzle precisely guides the material in a predefined path, forming a thin layer with a specified width. A typical extruder moves along the X and Y axes, and after each layer is printed, the building plate lowers by a specified height along the *Z* axis. The print head operates based on a specially generated file called G-code, which contains instructions for the movement path, printing speed, temperature settings, and extrusion commands [28,29,30].

The design of the target object, created in a CAD program, is exported as an STL file. STL is the most widely used format for representing a 3D model as a mesh of triangles and allows further modification or opening of the model in software designed for preparing the 3D printing process [31]. The built-in software integrated with a specific 3D printer is known as firmware. In these environments, users configure print preferences and parameters such as infill density, layer thickness, support structures, filament type, build volume, and extruder speed. This is also where the model undergoes the slicing process, which divides it into layers and enables export to a G-code file [28,29,30].

Various materials can be used for manufacturing objects with FDM technology. Most commonly, these are thermoplastic polymers such as polylactide (PLA)—widely used due to its low melting point and ease of printing, Acrylonitrile butadiene styrene (ABS), which is stiffer, more durable, but also more brittle, and flexible filaments, which offer elasticity such as polyurethane (TPU) [30,31]. In addition to standard polymers, FDM has also been adapted for use with ceramic pastes, as demonstrated by Antonio Hinojo et al. [32]. The polymer base can be modified in different ways, for example, to create conductive filaments that enable the printing of electronic components, circuits, or electrodes [33].

### 3.2. SLA—Stereolithography

The SLA technique is one of the Vat Polymerization Printing, which also consists of Digital Light Projector (DLP) or Liquid Crystal Display (LCD). The basic principle of VPP is light activated polymerization of different liquid resin monomers or oligomers. In the Stereolithography a laser beam moves along the programmed path curing the resin, and working place by place, pixel by pixel. This is what differentiates the SLA from DLP or LCD, which use light projected through a mask to cure the entire layer at once. While DLP and LCD are generally faster, they are also more complex to manufacture and therefore less widely adopted [34,35].

The earliest research on the use of photopolymers for creating 3D objects took place in the 1960s at the Battelle Memorial Institute in Ohio. The goal of the experiment was to polymerize the resin by intersecting two laser beams of different wavelengths. Later in 1971 Wyn Swainson filed a patent application for a similar method using two laser beams, which he called photochemical processing [36].

One of the first scientist to develop a rapid prototyping technique using a single laser beam was Hideo Kodama, from the Nagoya Municipal Industrial Research Institute in Japan [37]. Although he filed a patent for this invention in 1980, the application expired without progressing through the subsequent stages of the Japanese patent process. He published papers on his experiments to develop methods for the automatic fabrication of three-dimensional objects using UV rays and photosensitive resin, employing a mask to control exposure to the light source. He described techniques for curing thin, successive layers of photopolymer, which were key aspects of what later became known as stereolithography (SLA) [38]. A few years later, in 1986 Charles Hull, also interested in this research area, filled out the patent for the Stereolithography where this technique was described as the process of hardening liquid polymers layer by layer under the UV light. This patent lead Hull to founding 3D Systems, which resulted to first produced and commercialized stereolithography machines [39,40,41].

The materials used in this technique are various types of resins, typically composed of monomers, oligomers, photo initiators, and other additives. The specific formulation of a resin determines its properties and suitability for different applications. SLA resins generally offer a smooth surface finish, which can be further enhanced through post-processing. One of the main advantages of SLA printing is its high precision and the excellent quality of printed prototypes. Commonly used materials include thermosetting photopolymer resins with a range of properties—such as opaque, rigid, transparent, flexible, or heat-resistant. The SLA process enables the production of finely detailed objects with smooth surfaces and minimal stair-stepping effects [42,43].

SLA technology can be applied in a wide range of fields, including dental and biomedical applications. Biocompatible resins specifically designed for custom medical devices or prosthesis are readily available on the market [44,45],

One of the challenges for SLA applicability is resin formulation; however, recent research has led to the development of new formulations or the modification of existing ones to suit novel applications. Research has demonstrated the use of stereolithography in environmental photocatalytic applications, for pollutant degradation [46], biomaterials and medical applications [47,48]. Additionally, SLA has been explored in advanced technical ceramics, where optimized photo-reactive suspensions enable the precise printing and sintering of components made from materials like silicon carbide (SiC)—a significant breakthrough for aerospace and aviation industries [49]. SLA is also employed in the development of potentiometric sensors, both for fabricating sensor platforms [50] and for printing ion-selective membranes [51].

### 3.3. Polyjet 3D Printing

The first Polyjet Printing machines were launched in 2000 by Objet Geometries Ltd. (Rehovot, Israel) which in 2012 was bought by Stratasys. The Polyjet technique combines Inkjet printing with Ultraviolet polarization to achieve final product. This printing method utilizes a UV curable resin, which is jetted in a small droplet onto the substrate or a printing plate and cured simultaneously after dropping. The usage of light polymerization makes it like the SLA method, but it is not categorized as Vat Polymerization but rather as the Material Jeting. A common feature of both methods is the use of the same materials and the resulting similarity in the properties of the manufactured objects such as excellent surface finish and high precision [42,51].

We can diverse the process of PolyJetting into three following steps: pre-processing, processing, and post-processing. In the first stage the orientation of the part on the build plate is optimized, using the software. Next, in the processing part droplets of resin are jetted onto the tray via an extruder, each droplet or layer is simultaneously cured via a UV light source located next to the printing nozzle. The building plate, as in the other methods of 3D printing, can move in a *Z*-axis and due to that the whole object is manufactured layer by layer. Very often the Polyjet printing accommodates two types of materials, the material of the object and the supporting one. In the last step the support parts are removed leaving a final product. The advantages of this 3D printing method are its precision–the accuracy of the parts obtained are 0.1 mm, the height of each layer is up to 16 μm and the ability to print with different types of material in one print allowing for manufacturing objects in which certain parts exhibits distinct properties [52,53,54].

### 3.4. LCM—Lithography-Based Ceramic Manufacturing

Another technique utilizing the photopolymerization process is Lithography-Based Ceramic Manufacturing (LCM), developed by Lithoz (Wien, Austria) [55]. This method enables the 3D printing of highly detailed and high-resolution objects, which has been employed in research to create potentiometric sensors for measuring tritium in the high temperatures [56]. The LCM technology operates by polymerizing ceramic powder suspended in a photosensitive resin. An LED light source projects an image onto the resin, selectively curing layers to build the component step by step. This process creates what is referred to as a “green part”, which, as explained by Lithoz, is a composite of ceramic particles embedded in a photopolymer matrix that acts as a binder for the particles. Subsequently, the green part undergoes thermal processing: first, the photopolymer matrix is removed in a debinding step, followed by sintering to achieve the final density of the part [57,58].

### 3.5. SLM—Selective Laser Melting

Another 3D printing technique is Selective Laser Melting (SLM), which falls under the Powder Bed Fusion (PBF) category of additive manufacturing. In this process, a heat source selectively melts the powder material spread on the build platform. Unlike Selective Laser Sintering (SLS) that only sinter the material, SLM fully melts the powder into a liquid, which then quickly solidifies. After each layer is completed, a new layer of powder is deposited on the object and the laser once again selectively melts the designated areas. This process is repeated layer by layer to create a complete 3D object [24].

Materials commonly used in SLM include metals, polymers, ceramics, and various alloys such as stainless steel-based, titanium-based, aluminum-based, and cobalt-based alloys [59].

The surface finish, mechanical properties, and overall quality of parts produced with SLM are often comparable to those made with traditional casting methods. However, SLM offers the added advantage of enabling the production of highly complex geometries, making it particularly useful for rapid prototyping. Additional benefits of the SLM technique include reduced design constraints, lower material waste, and decreased costs associated with tooling, equipment, and manual labor. In comparison to conventional casting or forging methods, PBF techniques like SLM are especially suitable for manufacturing high-precision, intricate components [60,61].

## 4. Application of 3D Printing Techniques in Manufacturing of Potentiometric Sensors

Rapid prototyping due to its versatility is becoming quite a widely applicable technique in manufacturing potentiometric sensors. Various 3D printing techniques enable scientists and researchers to fabricate advanced structures such as platforms, electrodes, flow devices, and ion-selective membranes. Wide range of materials utilized in 3D printing techniques shows a potential for further improvements of their properties, developing new materials or adjusting the existing ones for the additive manufacture techniques. Presented below is a wide array of solutions utilizing 3D printing, systematically grouped by the employed printing technique [62].

### 4.1. Application of FDM—Fused Deposition Modeling in Potentiometric Sensors

In the research paper by Mathew Mc Cole and collaborators [63], a portable system for on-site detection of soil pH and potassium levels using 3D printed sensors was described. Their work aimed to present a low-cost sensor capable of analyzing soil nutrients without the need for sample collection. The sensor introduced by this team consisted of a PSoC4 microcontroller and innovative miniaturized ion-selective electrodes printed using 3D printing technology—FDM. In this paper, a 3D-printed ISE for K^+^ measurement was constructed, along with a pseudo-reference Ag/AgCl electrode. The 3-D models of the electrodes were designed using CAD SolidWorks (version 2021). The electrode was constructed as a head with a diameter of 5 mm and a thickness of 1.5 mm, with a cylindrical rod of 3 mm in diameter and 20 mm in length. The electrodes were printed using FDM technique and carbon black infused PLA (polylactide). Ion-selective membranes (ISMs) were applied to the produced 3D-printed electrodes (3DPE) and to create the Ag/AgCl reference electrode, silver electrodeposition was performed with further electroanalytic deposition of chloride. The electrochemical cell consisted of two 3D-printed measurement electrodes and a reference electrode connected to the microcontroller. The electrical potential difference between the measurement electrode and the reference electrode for both parameters—pH and K—was measured using a dual-channel differential analog-to-digital converter (ADC). The results were transmitted to an external laptop or mobile phone via USB. The electrode’s ability to measure pH and detect potassium ions was confirmed using known concentrations of pH and potassium ions, showing high selectivity and excellent sensitivity consistent with the Nernst equation for pH (61.05 mV/pH) and potassium (49.50 mV/decade) [63].

**Figure 2:**
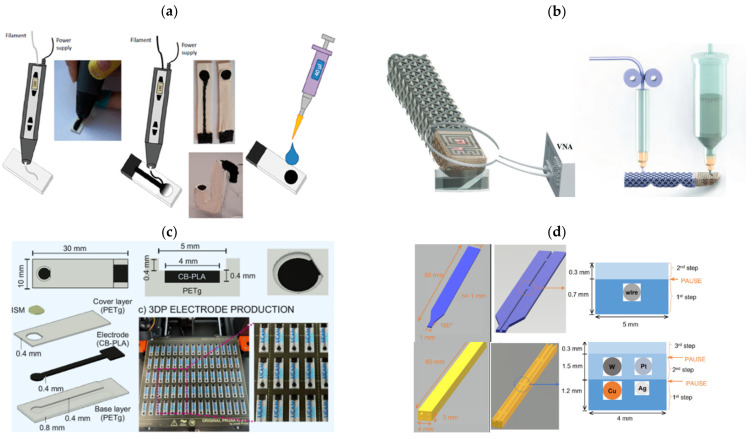

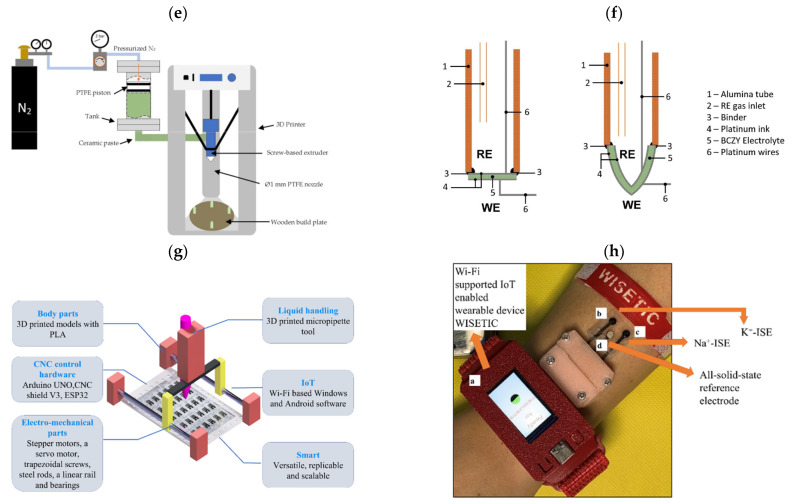
Diagram of various sensor designs and solutions aimed at optimizing processes using the Fused Deposition Modeling (FDM) technique: (**a**) Schematic representation of sensors preparation using a 3D pen: insulating layer prepared using a PLA filament, CB-PLA conductive track and support is drawn, membrane is drop cast, reprinted from [64]; (**b**) Experimental setup and printing sequence for wireless chemical sensing in a humanoid robotic hand reprinted from [65] with the permission of John Wiley & Sons; (**c**) Schematic of 3D-printed electrode and its layer by layer design reprinted from [33]; (**d**) Design of a single and multi-microelectrode body with a cross-sectional view of the channels containing the metallic electrode, reprinted from [66] with permission from Elsevier; (**e**) Schematic representation of the experimental setup used for printing of ceramic sensor reprinted from [32]; (**f**) Diagram of the potentiometric sensors constructed with BCZY electrolyte reprinted from [32]; (**g**) Schematic of a 3D printed micropipette robot and its key features reprinted from [67] with permission of Elsevier; (**h**) Portable Wi-Fi supported wearable device manufactured with micropipette robot, reprinted from [67] with permission from Elsevier.

Following a similar approach to the previously mentioned work, Justyna Kalisz et al. [64] utilized PLA combined with carbon black to develop potentially disposable potentiometric sensors. A 3D pen was used to shape the electrodes according to user-defined configurations as shown in Figure 2a. Ion-selective membranes were then applied onto the prepared electrodes. In their study, the researchers fabricated several electrodes sensitive to different analytes, including K^+^, Ca^2+^, and Cl^−^. For the K^+^-selective electrode, a linear relationship between the potential and the logarithm of K^+^ activity was observed within the range of 10^−1^ to 10^−7^ M, with a near-Nernstian slope of 55.5 ± 0.6 mV (R^2^ = 0.999). The 3D-printed Ca^2+^ ISE exhibited a slope of 24.6 ± 1.2 mV/decade (R^2^ = 0.995) and a detection limit of 10^−4.6^ M. For the Cl^−^ ISE, a linear response was obtained over the 10^−1^ to 10^−5^ M range, with a slope of −52.2 ± 0.3 mV/decade (R^2^ = 0.999) and a detection limit of 10^−5.2^ M. The tested electrodes demonstrated good potential stability, with standard deviations (SD) of the recorded potentials below 1.5 mV for the cation-selective electrodes and below 2.5 mV for the chloride-selective electrode. Additionally, the K^+^ electrode maintained a standard deviation of 1.5 mV over a five-day testing period [64].

The 3D printing approach of fabricating the electrochemical sensors was also utilized by H. B. Ho et al. [51] to propose a novel method for detecting of biomarkers of Parkinson’s disease. Parkinson’s disease cause an imbalance in acetylcholine an dopamine levels which can be monitored via potentiometric and voltametric techniques [51,68]. To print both potentiometric and voltametric electrodes, the researchers used a conductive PLA filament with Carbon Black purchased from ProtoPasta. The 3D printed carbon electrodes were fabricated using an FDM 3D printer. A CAD of a 5 mm diameter working electrode was uploaded to the 3D printer and 40 carbon electrodes were printed in ~15 min. For the ISEs, the ion-selective membranes were fabricated via Stereolithography as detailed in [69,70]. This 3D printing approach for constructing components of ISEs is further discussed in the next chapter. Interestingly, this research group has developed a Liquid contact ISEs (instead of solid contact) by using a small excess of resin used for ISM to bind it to PVC tubing. The inner solution was added (1 mM Ach^+^ and KCl in DI water) along with an Ag/AgCl wire and then conditioned in 10 mM Ach+ solution overnight (~14 h). The 3D-printed Ach^+^-ISE showed a linear Nernstian response with a slope of 56.4 mV/decade over a concentration range from 10 mM to 156 μM. The Ach+ sensor exhibited excellent stability, with an average drift of only 195 μV/h over a 12-h period. To facilitate the integration of both techniques (potentiometric and voltametric) into a device for simultaneous quantitative measurements of acetylcholine and dopamine at the POC, they designed a simple device. This device was printed using non-conductive filament via FDM 3D printing [51].

A more complex solution in regards of 3D printing was proposed in the research paper by Taeil Kim et al. [65]. In this study, FDM technology was used to print with different filaments—rigid and conductive—to create robotic hands capable of measuring multiple ions simultaneously. The system is equipped with an ISME sensor and an LC circuit that enables wireless data transmission. The ISME sensor and LC circuit were printed on opposite sides of a fingertip using ink printing, while the finger body and the fingertip were produced using dual FFF. A RostockMax SeeMeCNC^®^ printer, equipped with a four-nozzle extruder system, was used to fabricate the structure of the robotic finger. Semiflex was utilized for the finger body, while ABS and Electrifi were used for the fingertip. Finally, the palm was printed with PLA to connect the hand to the motor. The construction of the humanoid finger body and subsequent 3D printing of the integrated LC circuit are shown in Figure 2b. Each of the three fingers was designed to target a specific ion, allowing for the simultaneous measurement of three different ions, such as potassium, calcium, and ammonium. This study highlights the potential of using 3D printing to create more complex structures in a more efficient and affordable manner [65,71].

Two filaments used in one measure system were also used in the work of Daniel Rojas et al. [33]. The research group utilized in one print a conductive (PLA with Carbon Black by Protopasta) and nonconductive filament (PETg). In this study, an automated fabrication method for potentiometric SC-ISEs was developed based on FDM 3D printing for the measurements of K^+^ ions. This approach resulted in high electrode-to-electrode reproducibility, achieving a 0.5% RSD in E_0_. To achieve a one-step print of two materials with one nozzle the G-code was specially modified to allow filament changes at designated layers. This allowed the combination of PETg and CB-PLA to produce fully insulated, ready-to-use electrodes. A schematic of the electrode’s layered structure can be found in Figure 2c. The K^+^ selective membranes were fabricated by drop casting of the ionophore solution. The 3DP-SC-ISE exhibited nearly Nernstian behavior, with a slope of 57.7 ± 0.2 mV/decade, a linear detection range between 10^−5.5^ and 10^−2^ M, and a detection limit of 10^−5.9^ M. Importantly, excellent between-electrode reproducibility was achieved: RSD values were 0.2% for the slope and 0.5% (645 ± 3 mV) for E_0_. Compared to traditional GCE electrodes, which showed much greater variability (E_GCE_ = 630 ± 29 mV), the reproducibility was improved by an entire order of magnitude. The response time was also very favorable; the slowest response observed (at a half-decade concentration step) was just 9 s (t_95_%), which aligns well with expected potentiometric ISE standards. Method proposed in this study is highly cost-effective (approximately € 0.02 per sensor), enables fast production (75 electrodes in 210 min per printer), and is easily scalable, as affordable FFF printers are widely available (under € 200) [33].

In the work by Mariela Alicia Brites Helu et al. [66] a 3D printing process was used to create a body that enabled the construction of a multi-electrode system for potentiometric pH measurements and voltametric H_2_O_2_ measurements. The microelectrodes produced in this study were fabricated using FDM technology with PETg filament. The process involved printing an insulating body for the electrodes, inserting an etched metal or carbon rod, and then resuming the print to complete the sensor. The microelectrodes, composed of Pt, C, Au, Ag, W, and Cu with diameters below 5 μm, were fabricated and tested using cyclic voltammetry and scanning electron microscopy. Additionally, a multi-electrode probe composed of W, Cu, Ag (subsequently oxidized to Ag/AgCl), and Pt was designed, and 3D printed for potentiometric pH measurements and amperometric detection of H_2_O_2_, making it suitable for miniaturized sensing applications. The design and cross-sectional view of both single and multi-electrode probes are depicted in Figure 2d. Solid-state pH electrodes have been widely used due to their ability to operate at high temperatures. Common materials include iridium oxide [72,73] as well as tungsten and tungsten oxides [74,75]. In this study, a WO_x_ was used, with pH sensitivity arising from its proton-coupled redox reaction, following the Nernst equation. Open-circuit potential measurements were performed using a two-electrode setup: tungsten as the working electrode and Ag/AgCl as the reference. Pt and Cu electrodes were left unconnected. The system showed a strong linear correlation (R^2^ = 0.9988) between potential and pH (range 4–10), with a slope of −83 mV/pH. After completing the four-point calibration, three aqueous samples were tested: tap water, Milli-Q water and a commercial oral antiseptic solution. The results obtained with the fabricated electrodes were comparable to those measured with the commercial glass electrode with deviations under 0.5 pH units. The authors of the article also point out the relatively slow response time (around 100 s), mainly caused by the small dimensions of both the working electrode (WE) and the reference electrode (RE), which makes system depolarization more difficult. The work conducted by this group further demonstrates the practicality of 3D printing, especially in the field of manufacturing, by significantly reducing both production time and cost. During the study, they successfully produced forty-two electrodes in a single batch. This approach facilitates true miniaturization of the electrodes and greatly simplifies their preparation process [66].

The FDM method can also be used with materials other than conventional thermoplastic polymers. This idea was presented in the work by Antonio Hinojo et al. [32] in which potentiometric sensors were based on solid electrolytes with a perovskite structure. In this study, BaCe_0.6_Zr_0.3_Y_0.1_O_3_-α (BCZY) was used as a proton-conducting electrolyte for constructing potentiometric sensors capable of hydrogen (H_2_) measurements. This type of material is traditionally processed using uniaxial pressing. Two different shapes of sensor electrolytes were tested: pellets (BCZY-Pellet) and crucibles (BCZY-Crucible). These ceramics were formed using extrusion-based 3D printing. The setup used for ceramic sensor printing is represented in Figure 2e. Ultimately, parameters such as sensitivity, response time, recovery time, detection limit, and accuracy were evaluated for both sensor types (BCZY-Pellet and BCZY-Crucible) at a temperature of 500 °C. The sensors consisted of a working electrode, with the main component mentioned above-ceramic element-coated with a layer of platinum ink and a reference electrode, this setup is shown on Figure 2f. Those sensors demonstrated a linear response between 0.02 and 0.5 mbar and good repeatability. Measurements conducted with the sensors developed in this study were performed at 500 °C, showcasing their potential for applications in high-temperature conditions, such as hydrogen production processes [32].

In the work of T. Ozer et al. [67] they demonstrated a robot prototype to enhance reproducibility and reduce time of fabrication of ion-selective electrodes. The developed 3D printed robot controlled with internet of things (IoT) technology aimed to minimize human error during deposition of carbon black intermediate layers, ion selective membranes and reference membrane cocktails. The robot’s body parts were printed with PLA filament and Creality Ender 3 Pro FDM printer. A servo motor-driven tool was implemented and attached to the *Z*-axis to control the deposition process. To produce the membranes and conductive layers, G-code files were generated based on the screen-printed electrode designs, allowing for precise control of the glass vial and drop-cast solution. For IoT connectivity, a Wi-Fi-enabled microcontroller supporting relevant communication protocols was used. A diagram of the 3D-printed micropipette robot and its operating principle are presented in Figure 2g. This system facilitated accurate deposition of both carbon layers and ion-selective membranes, offering a fast, cost-effective, and error-free alternative to manual application and assembly of ISE components. To verify the accuracy and repeatability of layer deposition by the 3D-printed robot, screen-printed electrodes fabricated according to their previous study [76] were used. These were coated with a conductive intermediate layer and ion-selective membranes for K^+^ and Na^+^ ions. The actual reduction in defect rate, compared to manual drop-cast layers, was confirmed using SEM images. The results showed that surfaces of conventionally prepared ISEs exhibited scratches and bubbles, whereas those produced by the 3D-printed robot were defect-free. The potentiometric response was measured both in the solution of primary ions and in the artificial sweat solutions including all interfering metabolites including Mg^2+^, Ca^2+^, sucrose, glucose, HPO_4_^2−^, NO_3_^−^, and CO_3_^2−^ and resulted in stable response in the concentration range 10^−5^–10^−1^ M for both primary ions. The sodium ISE exhibited Nernstian responses of 58.2 ± 2.6 mV decade 1 and the potassium ISE showed 56.1 ± 0.7 mV decade 1, which is lower than the theoretical Nernst response but was explained by the similar behavior observed in other studies [77,78]. To test the stability of the developed ISEs and the solid-state reference electrodes, real-time monitoring was conducted over 3.5 h relative to a standard reference electrode. The observed changes in potentiometric response were 0.3 mV/h, 0.5 mV/h, and 1.2 mV/h, respectively. The reproducibility of the Na^+^-ISEs was assessed using the standard deviation of the standard potential (E^0^) for five individual electrodes, resulting in a value of 7.8 mV. Finally, to verify the practical applicability of the prepared electrodes, tests were conducted using artificial human urine and sweat samples. The results showed relative errors between 2.4% and 9.4%, indicating good measurement accuracy [67].

### 4.2. Application of SLA—Stereolithography Modeling in Potentiometric Sensors

**Figure 3:**
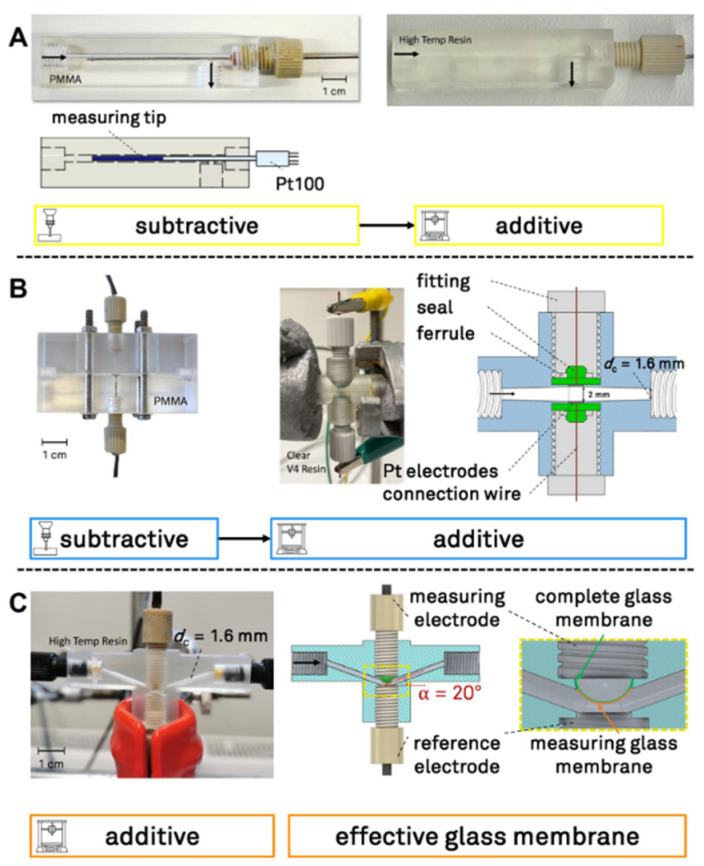
Overview of the construction of the microfluidic sensor flow cells measuring: (**A**) temperature, (**B**) electrical conductivity—EC, (**C**) pH value, reprinted from [81].

One of the approaches to applying potentiometric sensors is manufacturing a flow device, ready for in-line operation. Three-dimensional printing can be applied to print microfluidic cells, which serve as an alternative to commercial analytical devices [79,80]. In the study of R. Dinter [81], a constructed device enables simultaneous measurement of temperature, electrical conductivity, and pH value. In the study, a compilation of three sensor flow cells was developed and manufactured. The microchannels were first produced using additive manufacturing—SLA 3D printer, the Formlabs (Somerville, MA, USA) Form 3+, and photocurable resin–High Temp and Clear V4 Resin. Figure 3 illustrates the construction of microfluidic sensors, highlighting the differences between traditional subtractive manufacturing methods and additive (3D printing) approaches. The accuracy and precision of these flow cells were evaluated against commercial reference devices to determine the overall performance of the customized microfluidic flow cells. For accuracy testing, a 50 mL HCl solution was neutralized by the continuous addition of 1 mL/min NaOH, with the theoretical inflection point expected at pH 7. The study demonstrated that construction using 3D printer flow cell systems can reliably measure the pH value within the 2% margin of theoretical predictions, which is well within the typical range for flow titration. These findings suggest that the tailored microfluidic inline pH sensor is effective for real-time tracking of reaction dynamics, including conversion, activation energy, pre-exponential factors, and critical titration characteristics such as pE and pI [35].

In another study demonstrated by w. Lonsdale et al. [82] a fully solid-state potentiometric pH sensor based on a thin-film RuO_2_ working electrode was developed. The sensor design incorporated a RuO_2_ reference electrode modified with polyvinyl butyral-SiO_2_ and a 3D-printed electrode housing. The electrode housing was fabricated using light-cured acrylic resin (Peopoly) with a Peopoly Moai stereolithographic 3D printer. Several electrode housing designs were tested. For assembly, the RuO_2_ electrode was placed into platform, and electrical wires were inserted through openings and connected to the electrodes using carbon paste. The electrical contact was then waterproofed with the same Peopoly resin, cured using a portable UV light source [36]. The developed sensor demonstrated a linear pH response (−55.7 mV/pH, R^2^ = 0.9997) and satisfactory repeatability. Experimental results showed that using the previously developed calibration sampling protocol and single-point calibration, the sensor achieved higher accuracy compared to a commercial glass pH sensor [82].

The application of 3D printing was also presented in the work of Tao Zhang ad al. [83] for the construction of a potentiometric sensor capable of sensitive, accurate, and rapid detection of bacteria responsible for urinary tract infections. An Ag^+^ -sensitive electrode made using 3D printing technology—MultiJet Printing–was used to determine the number of bacteria. In this technique, multiple printing nozzles precisely deposit thin layers of photopolymer material, which are cured with UV light. Silver ions were absorbed by the bacteria, and by measuring changes in the concentration of Ag in the samples, it was possible to estimate the number of bacteria. In addition to the electrode, a 3D-printed filter was also created to avoid Cl- interference in urine, which could cause Ag^+^ precipitation and disrupt the measurement. The filter enabled quick separation of bacteria from the urine matrix and was printed in two parts, one using MultiJet Printing method and the other using typical stereolithography [83]. To characterize designed electrodes, four solutions were prepared with different concentration of AgNO_3_ and the calibration process was completed. From the commercial electrode the calibration slope was 65.3 mV and from the 3-D printed one–61.8 mV, while the theoretical slope based on the Nernstian equation should be 59.2 mV. The 3D printed electrode varied in slope by 4.4% while the commercial one by 10.3%. The response time measured between immersing electrode in the sample and a stable response was 10 s, which was lower than for the commercial one —30s. The construction of the device allowed for the minimizing of the sample amount from 3ml for the commercial one to less than 300 μL. This study also demonstrates that 3D-printed electrodes can be fabricated at a significantly lower cost than standard commercial electrodes, with the designed electrode costing less than $10 compared to over $200 for commercial alternatives. Urine analysis is a simple and non-invasive approach to diagnose and monitor various health disorders. Although urine analysis is primarily limited to clinical laboratories, the non-invasive collection of samples makes it applicable in a wide range of settings beyond central laboratories. In this context, devices capable of measuring multiple parameters simultaneously and operating at the point of care could have widespread use [84].

In the study by M. Dębosz et al. [85] a flow manifold was developed for the determination of sodium, potassium, and calcium ions in urine samples using ion-selective electrodes based on solid contacts. Solid-contact designs enable miniaturization and, in the context of 3D printing, offer significant design flexibility and customization of devices for specific applications, making 3D printing one of the most cost-effective solutions for ensuring broad accessibility [86]. This study evaluated a system for multiplexed determination of sodium, potassium, and calcium ions in urine samples, using ion-selective electrodes based on modern solid contacts with multi-walled carbon nanotubes functionalized with octadecylamine (OD-MWCNT) [85,87]. Used above electrodes were manufactured following the procedure described in another work by Dębosz [88] where the flow device was constructed for the measurement in water. The flow housing was produced using stereolithography and 3D printers (Formlabs) with Dental Clear Resin (Formlabs). A gold wire was placed inside the electrode housing, and the space between the housing and the wire was sealed with quick-drying epoxy resin. The electrodes were tested within a clinically relevant concentration range, approximately 10^−4^ to 10^1^ mol L^−1^ of each ion, and demonstrated near- or Nernstian responses under flow injection conditions [85,88]. The slope of the calibration curve was as follows for K^+^ ISE: 59.5 ± 0.6 mV/dec, Na^+^ ISE: 55.7 ± 0.6 mV/dec and Ca^2+^ ISE: 27.3 ± 0.6 mV/dec. Besides obtaining the calibration curves, the applicability of the flow injection system was verified through the analysis of synthetic samples and two certified reference materials (CRM) for urine. The accuracy of the obtained measurements had the relative standard deviation not exceeding 4.0% and the relative error ranging between 8.0% and 6.0% in comparison to the real concentration of the tested artificial-clinical samples.

In the previously mentioned article [88] the group led by M. Dębosz developed a 3D-printed flow manifold for the simultaneous measurement of multiple ions—potassium, sodium, calcium, and chloride—in water samples. The method relied on the use of miniaturized solid-contact ion-selective electrodes (ISEs) with a specialized design achieved through 3D printing. One of the key advantages of using a specially designed 3D-printed flow cell for potentiometric measurements was the miniaturization of electrodes and the ability to integrate several (from three to six) ion-selective electrodes into a single module. This enabled multi-component analysis to be conducted simultaneously, significantly reducing the sample volume and measurement time required for such analyses. The ISE housings were fabricated using a stereolithographic 3D printer (Form 2, Formlabs). In optimization studies, two types of materials were tested for electrode housing: Standard Resin (Formlabs) and Dental LT Clear (Formlabs). These materials were chosen not only due to the technical constraints of the printer but also for their durability and chemical resistance. Importantly, the selected materials do not dissolve in tetrahydrofuran (THF), which is used in the preparation of ion-selective membranes [88]. To evaluate the applicability of the measurement system, the same analytical method used for urine samples was applied to synthetic water solutions. Three synthetic samples were prepared containing calcium, sodium, and potassium ions at concentrations of 1.00, 5.00, and 25.00 mmol L^−1^, and chloride ions at concentrations of 4.00, 20.00, and 100.00 mmol L^−1^. All four analytes were analyzed simultaneously using the developed system. The results showed relative errors of less than 8%, and the relative standard deviation did not exceed 3%, demonstrating the method’s reliability and precision for multi-ion detection [88].

Wearable sensors have recently gained significant popularity due to their ability to continuously measure various parameters. These devices can be highly beneficial for diagnostic or therapeutic purposes, such as monitoring biomarkers or for minimizing the inconvenient and painful sampling for the diabetes patients [89]. In the study by Marc Parilla [50], 3D printing technology was employed to fabricate sensors based on microscopic needles (MNs), which allowed access to interstitial fluid (ISF). The use of 3D printing with low force stereolithography enabled resolutions down to the micrometer scale. To facilitate potentiometric measurements, the fabricated patches were modified with conductive inks. The hollow needles filled with conductive ink functioned as microelectrodes, enabling in vitro pH measurements over a wide range. The working and reference electrodes consisted of modified polyaniline and polyvinyl butyral, respectively. The MN-based sensor was tested on a human forearm to evaluate its real-time monitoring capabilities [50]. To evaluate the repeatability, stability, reversibility and to construct the calibration curve a set of Briton-Robinson buffer solutions from pH 4 to 10 was prepared. To calculate the selectivity coefficient a certain amount of interferent was added into the pH solutions. To evaluate the sensor’s applicability for measuring interstitial fluid, a synthetic gel mimicking skin was prepared following a procedure similar to that reported in the literature [90], with a composition based on actual sample data [91]. Additionally, clinical tests on the body were conducted.

The calibration curves were conducted using the buffer solution depicts a near Nernstian response of the 3D printed micro-needle system with the slope of 66.7 ± 4.1 mV/pH with an intercept of 595.4 ± 17.1 mV that depicts the reversibility of the pH monitoring. The on-body test was performed to analyze the pH value of the interstitial fluid. For the health subject the expected pH value should be equal to 7.4. The result of MN sensor was pH 7.56 ± 0.02 when not accommodating the temperature effect and pH 7.39 ± 0.01 when temperature factor was taken into consideration. The errors from the expected potential value (in pH 7.4) were 7.04% and 0.32%, respectively.

Using SLA method, it is possible to fabricate not only electrodes or sensor housing but also to prepare Ion Selective Membranes (ISMs). The ISM is critical component of potentiometric sensor that facilitates ion transfer across the membrane-solution interface while providing the electrode/system with its selectivity [92]. In conventional ISMs the primary components are a matrix (supporting material), a plasticizer, and an ionophore—a lipophilic compound capable of selectively binding targeted ions. The most commonly used matrix is polyvinyl chloride (PVC) [93,94,95]. The plasticized PVC membranes which were introduced more than two decades ago [4,11,12] dominate among similar materials, though silicone rubbers, photocured films and some natural polymers (like Urushi) also presents unique advantages [96]. As an alternative for PVC matrices, polyurethanes (PUs) has drawn particular attention, especially due to their suitability for biomedical applications involving all-solid-state or miniaturized in vivo ion sensors [97,98]. Some polyurethanes such as Tecoflex and Pellethane are valued not only for the manufacturing of implantable medical devices but also for formulating ion-selective membranes (ISMs) [99,100].

The preparation of ISMs using 3D printing methods has been explored by Nguyen H. Bo [68], whose fabricated electrodes with 3D-printed ISMs were applied for the detection of biomarkers of Parkinson’s disease [51] and benzalkonium chloride [70]. While the FDM technique was used for constructing the electrodes (as described previously), the focus here is on the fabrication of the ISMs. In both applications, the conventional PVC matrix was replaced with a commercial flexible UV resin and NPOE which served as the plasticizer. For the preparation of the 3D printable ISM cocktails, commercial flexible UV resin (80A, Formlabs) was used to substitute PVC. In the Ach^+^-selective membrane, NPOE was employed as the plasticizer, KTCPB as the ion-exchanging salt, and C4X as the ionophore, maintaining a 1:3 molar ratio with the salt. The Ach^+^-ISM cocktail composition was 96.0 wt% UV resin, 2.0 wt% NPOE, 0.5 wt% KTCPB, with the appropriate amount of C4X. Similarly, for the benzalkonium chloride-selective membrane, the cocktail was composed of 96.2 wt% UV resin, 2.0 wt% NPOE, 0.5 wt% KTCPB, and 1.3 wt% of the ionophore C6X (1:2 molar ratio with KTCPB). The CAD models for the ISM fabrication were identical for both applications; they were uploaded into the 3D printer’s software, printed using an SLA 3D printer (Elegoo 2 Pro), and post-processed by washing the printed membranes with isopropanol (IPA) to remove uncured resin, followed by rinsing with deionized water. Additionally, for the preparation of 3D-printed liquid-contact ISEs (LC-ISEs), a small excess of the flexible resin was applied onto the membranes to bind them securely to PVC tubing, followed by post-curing under a UV lamp for 10 min. This innovative approach highlights the potential of 3D printing technologies in advancing the fabrication of ion-selective electrodes with new material combinations and architectures. In both experiments, a carbon electrode fabricated by FDM 3D printing was used in combination with an Ag/AgCl wire serving as the reference electrode [51,70]. By incorporating the ionophore calix [6] arene, the 3D printed BA^+^-ISE exhibited excellent selectivity against common cationic ingredients of ophthalmic solutions and showed a linear Nernstian response, with a slope of 55 mV/decade, between 1 mM and 31 μM–covering the typical concentration range of Benzalkonium BA^+^ in eye drops. Additionally, the sensor displayed impressive stability, with an average potential drift of approximately 205 μV/h over a 14-h period. This stability and selectivity suggest that the 3D printed BA^+^-ISE would be capable performing continuous monitoring of BA^+^ in complex pharmaceutical formulations [70]. Similarly, the 3D-printed Ach^+^-ISE showed a linear Nernstian response with a slope of 56.4 mV/decade, covering a range between 10 mM and 156 μM, and demonstrated high stability with an average drift of 195 μV/h over 12 h [51].

Another study on the development of 3D printed ion selective electrodes was conducted by the team M. Mamaril et al. [101]. This research focused on the point of care (POC) diagnosis of hypocalcemia in dairy cattle through the detection of Ca^2+^ ions in biological samples. Ion–selective membranes were fabricated using SLA printer, an UV-curable resin, a plasticizer, the ion-exchange salt KTClPB, and the ionophore Calcium IV. A computer aided design (CAD) was uploaded to the 3D printer, and the ISMs were then printed with a diameter of 10 mm and a thickness of 200 μM. The Ca^2+^-ISEs were assembled by applying a small amount of excess resin to bond the ISM to PVC tubing, followed by post-curing under a 365 nm UV lamp for 5 min. The ion-selective membranes produced via 3D printing were integrated with paper-based sensors to demonstrate their potential for point-of-care diagnostics. For comparison, the team also prepared a classical sensing system with an internal solution. This traditional sensor exhibited a Nernstian response of 28 ± 2 mV/decade over a concentration range of 100 mM to 97.7 μM Ca^2+^ at pH 7.4. The paper-based sensor demonstrated a similarly linear calibration with a Nernstian response of 29 ± 1 mV/decade over the range of 10 mM to 100 μM, effectively covering both normal physiological and hypokalemic levels of Ca^2+^ in dairy cattle. To assess the sensor’s real-world applicability, its performance was compared with that of the commercially available epoc^®^ POC analyzer, commonly used by veterinarians. Blood samples were collected from local dairy cattle, and results obtained using the fabricated paper-based sensors were comparable to those from the epoc^®^ analyzer. For all three measurements, the results were within 5% of each other, with an average percentage difference of just 2.18% [101].

### 4.3. Application of LCM—Lithography-Based Ceramic Manufacturing in Potentiometric Sensors

To develop a sensor sensitive to H_3_, perovskite-type ceramics previously discussed in [102]—BaCe_0.6_Zr_0.3_Y_0.1_O_3_-α (BCZY)—was utilized. BCZY exhibits high proton conductivity and exceptional stability, even in reducing atmospheres and elevated temperatures, making it a strong candidate for hydrogen detection at high temperatures. In the study by A. Hinojo the development of hydrogen sensors was constructed using the LCM method. The crucible geometries were evaluated by X-ray diffraction (XRD) and scanning electron microscopy (SEM). The sensors were prepared for the application in higher temperature w range 400–500 °C, working in two modes–the amperometric and potentiometric one. The preparation of the ceramic suspension and the printing process were carried out by Lithoz GmbH in Vienna. The results demonstrated the ability of 3D-printed BCZY sensors to detect hydrogen [56]. When operated in potentiometric mode, the sensor functioned as a concentration cell, with the working electrode exposed to varying hydrogen partial pressures ranging from 0.020 to 1.000 mbar H_2_ in an argon atmosphere, while the reference electrode was maintained at a constant 1.000 mbar H_2_ in Ar. The difference in hydrogen partial pressure between the two electrodes generated a potential difference, consistent with the Nernst equation, which describes the electrochemical response based on the logarithmic ratio of hydrogen activities at each electrode. The sensor demonstrated useful linear range at three tested temperatures 400 °C, 450 °C and 500 °C with deviations from the theoretical Nernstian slope not exceeding 3.9%. Within the linear range, the correlation coefficients (R^2^) between the measured potential difference (ΔE) and the natural logarithm of hydrogen partial pressure (ln_(pH2)_) exceeded 0.99 confirming strong linearity. In a separate experiment, the sensor’s accuracy and precision were evaluated in the lower part of the linear range (0.020–1.000 mbar). Across all three temperatures, the coefficient of variation (CV) ranged from 0.8% to 1.1%, well within the accepted range of <4% [56,103].

### 4.4. PolyJet Printing in Potentiometric Sensors

**Figure 4:**
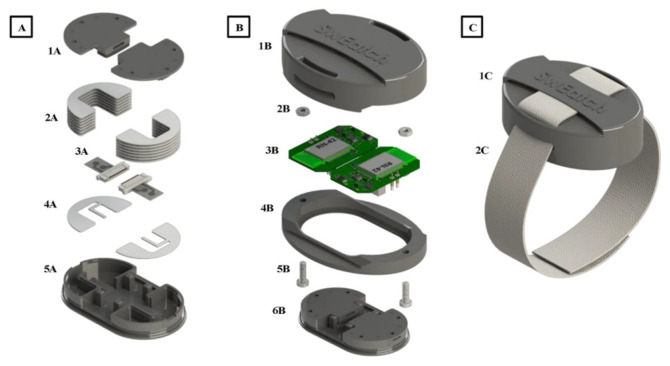
Overview of the construction of the 3D printed microfluidic unit in the form of wearable sensor: (**A**,**B**)—construction of the sensor, (**C**)—the final device [78].

In the work of Paolo Pirovano et al. [78], another 3D printing technique—PolyJet printing—was employed. The printing technique enabled the fabrication of structures composed of both rigid and flexible materials within a single object. Using this approach, the research team developed a redesigned housing for a sweat-analysis platform, building upon their earlier work [104]. The new design integrated a dual microfluidic component for simultaneous monitoring of two electrolytes in sweat in addition to two ISEs. The 3d printed platform featured a modular, three-part assembly that allowed for easy replacement of electronic components, electrodes, and absorbent materials during use. The platform was divided into three main units: a microfluidic module, a central platform body and the complete wearable system. The microfluidic component included a dual macro-duct and a two-half cylindrical sweat reservoir configuration enabling separate sample channels for sodium and potassium sensing. The redesigned microfluidic module improved contact between electrodes and the fluid channel’s capillary flow. Multi-material 3D printing allowed for better sealing and a more precise interface between the microfluidic unit and the platform body. To maintain stable skin contact during use, the device was secured with a strap threaded through three strategically placed loops on the top and sides. This ensured consistent positioning throughout on-body testing. The integration of this 3D-printed housing with screen-printed electrodes was successfully demonstrated in real-life sweat monitoring applications, which will be discussed in greater detail in a subsequent section of this article [78]. The construction of the final wearable device is shown in Figure 4.

### 4.5. Application of SLM—Selective Laser Melting in Potentiometric Sensors

**Figure 5:**
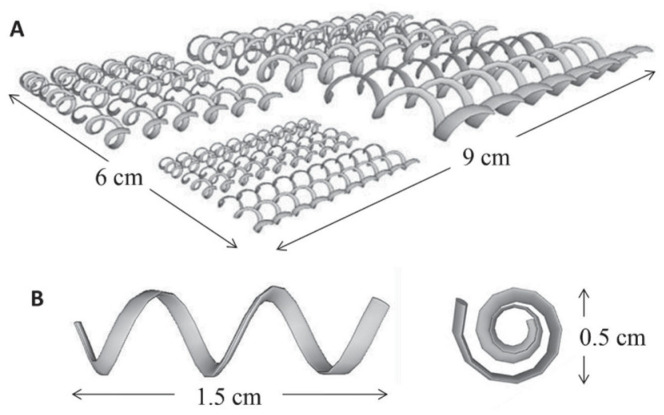
(**A**) Schematic of the electrode design used to print helical-shaped stainless steel electrodes; (**B**) Dimension of the electrodes employed in this work for the electrochemical testing reprinted from [105] with the permission of John Wiley & Sons.

The Selective Laser Melting (SLM) technique was utilized by Ambrosi et al. [105] to developed a potentiometric pH sensor based on a 3D-printed stainless-steel (3DP-SS) helical electrode, which was modified with electrochemically deposited IrO_2_ layer. This sensor exhibited excellent proton detection properties across a wide pH range (2 to 12) while adhering to Nernstian behavior. The 3DP-SS electrode was first modeled in an open-source software (Sketch-up version 2015), and the metal 3D printing was performed with a Concept Laser (Concept LaserGmbH, Lichtenfels, Germany) printer employing a SLM method. In this process, high energy laser beam fuses and melts the metallic particles in the form of powder deposited on the printing bed in accordance with the uploaded model. The electrode design, along with their respective diameters, is shown in Figure 5. While the conductivity of the steel electrodes made by using a 3D printer was comparable to the bulk metal conductivity, their practical performance was initially limited by high charge transfer resistance and slow electron transfer in solution. To address this, conductive coatings such as polypyrrole [106], polyaniline [107], or metal oxides like RuO_2_ [108] and IrO_2_ were considered [109,110]. Iridium dioxide was selected due to its well-known redox behavior and proven applicability in open-circuit potentiometric pH sensing. In the final design, the 3DP-SS electrodes were coated with IrO_2_, resulting in a sensor that demonstrated high linearity with pH changes and a slope value near the theoretical Nernstian response of 60 mV pH^−1^ [105].

## 5. Discussion

Table 1 presents a comprehensive overview of recent developments in the use of 3D printing and related additive manufacturing techniques for the fabrication of potentiometric sensor components. Various methods, including fused deposition modeling (FDM), stereolithography (SLA), screen-printing, PolyJet, and selective laser melting (SLM), have been applied to create functional elements such as working electrodes, reference electrodes, electrode housings, microfluidic cells, and wearable systems. The materials used range from conductive carbon-based PLA filaments and photocurable resins to advanced perovskite-type solid electrolytes. The table highlights not only the printing techniques and materials employed but also the specific target analytes—such as pH, potassium, sodium, calcium, acetylcholine, and hydrogen—demonstrating the versatility of additive manufacturing in electrochemical sensing. This comparison emphasizes the growing trend of integrating low-cost, customizable fabrication methods with classical electrochemical systems to produce sensors suitable for both laboratory and real-world applications.

**Table 1:** Overview of 3D printing and related additive techniques used in potentiometric sensor fabrication including printing material, printed part and detected analyte.

L.p	Printing Technique	Printing Material	Printed Part	Analyte	Reference
1	FDM	Carbon black infused PLA	Working Electrode	pH and potassium	[63]
2	FDM-3D pen	Carbon black infused PLA	Working Electrode	potassium, calcium, and chloride	[64]
3	FDM	Carbon black infused PLA-ProtoPasta	Working Electrode	Acetylocholine	[51]
4	FDM and Direct Ink Writing	Semiflex, ABS, Electrifil, PLA for Robot finger and Cellulose-based conductive Ink for the LC circuit	Robotic finger and LC circuit	Potassium, Calcium and Ammonium	[65]
5	FDM	PLA with Carbon Black and PETg	Fully insulated Working Electrode	Potassium	[33]
6	FDM	PETg	Insulating body for Microlectrodes	pH	[66]
7	FDM/DIM	Solid electrolytes with a perovskite structure-BaCe_0.6_Zr_0.3_Y_0.1_O_3_-α (BCZY) and Platinum Ink	Working Electrode	Hydrogen in high temperature	[32]
8	FDM	PLA	Robot for carbon layers and membrane deposition	Potassium and Sodium	[67]
9	SLA	Photocurable resin	Flow cell	pH	[81]
10	SLA	Photocurable resin	Electrode housing	pH	[82]
11	MJP and SLA	Photocurable polymer	Electrode housing and a filter for Cl interference ions	Ag+-detection of bacteria	[83]
12	SLA	Photocurable resin	Flow cell	Sodium, Potassium and Calcium	[85]
13	SLA	Photocurable resin	ISE housing	Sodium, Potassium and Calcium	[88]
14	SLA	Photocurable resin	Microneedles as Working Electrodes	pH	[50]
15	SLA	Photocurable resin with Ach+-selective ionophore or Benzalkonium Chloride Ionophore	ISM	Acetylocholine and benzalkonium chloride	[68]
16	SLA	Photocurable resin with Calcium Selective Ionophore	ISM	Calcium	[101]
17	LCM method	BaCe_0.6_Zr_0.3_Y_0.1_O_3_-α (BCZY)—	Working Electrode	Hydrogen in high temperature	[102]
18	PolyJet printing	Rigid polymer Veroblack, Flexible polymer TangoBlack	Housing of wearable sensor	Sodium and Potassium	[78]
19	SLM	Stainless steel	Working Electrode	pH	[105]

The application of 3D printing in the fabrication of potentiometric sensors has introduced several significant advantages that are reshaping sensor development. One of the most notable benefits is the ability to achieve high levels of miniaturization. Additive manufacturing techniques, such as fused deposition modeling (FDM) and stereolithography (SLA), enable the creation of compact, intricate geometries that are well-suited for wearable, implantable, or portable analytical devices. This capability also facilitates the integration of sensors with microfluidic structures, housing components, or connectors, all in a single print. In addition, the relatively low cost of desktop 3D printers and materials—particularly FDM filaments—makes this technology accessible for both research and scalable production. Rapid prototyping is another key strength; digital models can be quickly modified and reprinted within hours, enabling fast iteration during sensor optimization. Multimaterial printing, especially with conductive filaments or resins, allows simultaneous fabrication of both sensing and insulating elements, reducing assembly time, and enhancing sensor integration. Overall, these features promote flexibility, affordability, and innovation in the design and deployment of potentiometric sensors [111,112]. As described, the use of 3D printing in potentiometric sensor development offers several distinct advantages: (i) rapid and cost-effective prototyping, (ii) high degree of miniaturization, (iii) freedom in geometric design, and (iv) integration of multiple sensor components in a single step. Finally, the compatibility of 3D printing with wearable formats and microfluidic systems opens new possibilities for personalized, portable, and point-of-care sensing solutions. Together, these advantages highlight the transformative potential of 3D printing in modern electrochemical sensor technology.

Despite its advantages, 3D printing also presents several challenges that must be addressed for reliable potentiometric sensor development. One of the primary issues is reproducibility: sensor properties such as electrode resistivity, membrane adhesion, and signal stability can vary significantly between prints, even when using the same design. This variability is often influenced by printing parameters, including layer height, nozzle temperature, print orientation, and post-processing conditions. Moreover, materials commonly used in FDM (e.g., PLA, ABS, carbon-loaded filaments) and SLA (e.g., photocurable resins) may suffer from chemical instability, poor long-term resistance to aqueous solutions, or incompatibility with ion-selective membranes. For instance, printed electrodes can degrade or swell when exposed to certain solvents or electrolytes, leading to signal drift or membrane delamination. Another concern is the inherent surface roughness and anisotropy of FDM-printed parts, which can increase interfacial resistance and compromise membrane uniformity. SLA offers higher resolution but may introduce toxicity or require complex post-curing procedures. Lastly, the lack of standardized methods for integrating membranes and biological elements with 3D-printed substrates further complicates sensor consistency and long-term reliability. These challenges highlight the need for improved materials, optimized printing protocols, and careful post-processing to ensure the analytical performance of 3D-printed potentiometric sensors [112,113,114,115].

## 6. Future Perspectives

The future of 3D-printed potentiometric sensors lies in further integration, smart functionality, and enhanced material performance. One promising direction is the seamless merging of sensor elements with microfluidic systems and solid-state electronics. For example, recent advances demonstrated a fully 3D-printed Mg^2^-selective sensor incorporated into a microfluidic device, offering robust performance in low-volume biological samples and exceptional stability (~13 μV/h drift) [116]. This indicates that next-generation point-of-care platforms could be produced in a single manufacturing step, reducing assembly complexity and logistics.

Another major opportunity resides in novel multifunctional materials and 4D-printing approaches. Emerging photocurable resins and conductive composites tailored for additive manufacturing promise improved performance and biocompatibility. Advanced strategies, such as machine-learning-guided design of porous conductive architectures, are already enabling hierarchical electrode optimization for sensing applications. Meanwhile, 4D printing—where sensors can change shape or conductivity in response to environmental stimuli—holds potential for adaptive and implantable biosensing systems, although material development and stability under physiological conditions remain key challenges.

To advance this field, thorough work is needed on long-term durability, material compatibility, and standardization of fabrication protocols. Carefully engineered composites such as carbon-nanomaterial-loaded filaments require tailored treatment—e.g., laser or chemical activation—to achieve reliable conductivity and surface uniformity [62]. Additionally, scalable manufacturing demands printable inks that maintain performance over time and environmental exposure. This requires deeper understanding of interfacial chemistry, mechanical robustness, and sensor membrane integration.

To summarize, the road ahead for 3D-printed potentiometric sensors points toward fully integrated, responsive devices made from purpose-designed materials. Key milestones include developing stable conductive composites, adopting adaptive 4D structures, ensuring biocompatibility for clinical use, and embracing data-driven design. Overcoming these challenges promises a new era of personalized, digital electrochemical sensing with unprecedented versatility and manufacturability.

## 7. Conclusions

The integration of 3D printing technologies into the fabrication of potentiometric sensors represents a significant advancement in the field of analytical chemistry. Additive manufacturing methods such as FDM and SLA enable rapid prototyping, cost-effective production, and high design flexibility, which are particularly valuable for developing miniaturized, portable, and customizable sensing platforms. The ability to fabricate entire sensor assemblies, including housing, electrodes, and microfluidic components—within a single manufacturing process opens new possibilities for personalized and point-of-care devices.

Despite these promising developments, several challenges remain. Issues related to print-to-print reproducibility, material stability in complex chemical environments, and compatibility with ion-selective membranes must be carefully addressed. Moreover, standardization of printing parameters and post-processing procedures is essential to ensure consistent sensor performance and long-term reliability.

Ongoing advances in printable materials, multimaterial printing, and hybrid manufacturing approaches hold strong potential to overcome current limitations. As research continues to address these challenges, 3D printing is poised to become a mainstream tool for the design and implementation of next-generation potentiometric sensors in biomedical, environmental, and industrial applications.

## Abbreviations

The following abbreviations are used in this manuscript:

3DPE	3D-printed electrode
3DP-SS	3D-printed stainless-steel
ABS	Acrylonitrile butadiene
Ach+	Acetylocholine
AOA/OA	antioxidant/oxidant activity
ASS-ISE	all-solid-state ion-selective electrode
BA+	Benzalkonium
BCZY	BaCe_0.6_Zr_0.3_Y_0.1_O_3_-α
BJ	Binder Jetting
CAD	Computer Aided Design
CAM	Computer Aided Manufacturing
CB-PLA	Carbon Black with PLA
CRM	certified reference materials
CWE	Coated wire electrode
DED	Directed Energy Deposition
DLP	Digital Light Projector
FDM	Fused deposition modeling
FFF	Fused Filament Fabrication
HPM	Hybrid Potentiometric Measurements
IoT	Internet of Things
ISE	ion-selective electrode
ISF	interstitial fluid
ISM	ion-selective membrane
ISME	Ion-selective Membrane Electrode
LCD	Liquid Crystal Display
LC-ISE	Liquid-Contact ISE
LCM	Lithography-Based Ceramic Manufacturing
ME	Material Extrusion
MJ	Material Jetting
MN	Microneedles
OD-MWCNT	multi-walled carbon nanotubes functionalized with octadecylamine
PBF	Powder Bed Fusion
PEDOT	poly(3,4-ethylenedioxythiophene)
PET	polyethylene terephthalate
PETg	Polyethylene Terephthalate Glyco
PGA	Porous Graphene Gel
PLA	Polylactide
POC	Point of care
POT	poly(3-octylthiophene-2,5-diyl) (POT).
POU	Point of use
PPy	Polypyrolle
PU	Polyurethane
PVC	polyvinyl chloride
QRE	Quasi Reference Electrode
RE	Reference Electrode
RGO	Reduced graphene oxide
SC-ISE	Screen Printed Ion Selective Electrode
SL	Sheet Lamination
SLA	Stereolithography
SLM	Selective Laser Melting
SLS	Selective Laser Sintering
SPE	Screen Printed Electrode
THF	tetrahydrofuran
TPU	polyurethane
VP	Vat Photopolymerization
WE	Working Electrode
